# Effect of repeated intravitreal anti-vascular endothelial growth factor drugs on corneal nerves

**DOI:** 10.1097/MD.0000000000034210

**Published:** 2023-07-21

**Authors:** Yuanyuan Qi, Lin Cui, Li Zhang, Chunxiao Yan, Yao Jiang, Shuang Ye, Lili Ji, Yuanyuan Qiu, Lijun Zhang

**Affiliations:** a Department of Ophthalmology, the Third People’s Hospital Affiliated to Dalian Medical University, Dalian, China; b Key Laboratory of Corneal and Ocular Surface Diseases Research of Liaoning Province, Dalian, China; c Dalian Medical University, Dalian, China.

**Keywords:** aflibercept, anti-vascular endothelial growth factor, confocal microscope, cornea, nerve density, nerve length, ranibizumab

## Abstract

To investigate the potential effect of repeated intravitreal injection of anti-vascular endothelial growth factor (anti-VEGF) drugs on corneal nerves. A total of 64 patients were treated with intravitreal injection of anti-VEGF drugs. There were 19 cases of neovascular age-related macular degeneration (AMD), 20 cases of diabetic macular edema (DME) and 25 cases of retinal vein occlusion (RVO). Twenty-nine cases were treated with aflibercept (2 mg/0.05 mL) whereas 35 cases were managed with ranibizumab (0.5 mg/0.05 mL). A corneal confocal microscope was used to collect images of corneal subbasal nerve plexus, and Image J was used for image analysis. The changes in corneal nerve were compared between 1 month after each injection and before injection. There were no significant differences in the density and length of corneal nerve at specific time after the surgery in comparison with baseline in patients who were given 3 intravitreal injections. There was no significant correlation between the numbers of injections and the changes of the corneal nerves. After 3rd injection, the nerve length of the DME group was markedly lower than that of AMD and RVO groups, the difference was statistically significant (*P* < .05). The nerve density of the DME group was not significantly different from that of AMD and RVO groups, whereas the nerve length and nerve density of the AMD and RVO groups were not statistically significant between each other also. The corneal nerve length after the 2nd and 3rd injections of Aflibercept were lower than that before surgery, the difference was statistically significant. There were no significant differences in nerve density and nerve length at each time point after Ranibizumab injection. The length and density of the corneal nerve after multiple injections in contralateral eye displayed no significant changes compared with the baseline. Repeated intravitreal anti-VEGF drug can reduce the length of corneal nerves. For patients who need repeated intravitreal injections of anti-VEGF drugs, especially in DM, attention should be paid on the changes affecting the corneal nerves. It is also needed to strengthen the local anti-inflammatory therapy to avoid infection and to use artificial tears to protect the microenvironment of the ocular surface after the surgery.

## 1. Introduction

Since 2006, intravitreal injection of anti-vascular endothelial growth factor (anti-VEGF) drugs are being used to treat neovascular age-related macular degeneration (nAMD),^[1]^ diabetic macular edema (DME),^[2]^ retinal vein occlusion (RVO).^[3]^ Repeated intravitreal anti-VEGF drugs are required to significantly improve the visual acuity and visual distortion and are widely concerned by ophthalmologists and patients. Ocular complications caused by the repeated injections have emerged a critical issue of widespread concern. Studies have found that VEGF factors and anti-VEGF drugs can be detected in aqueous after intravitreal injection of anti-VEGF drugs,^[4]^ and VEGF and its receptors can also be expressed in corneal epithelium and corneal endothelial cells.^[5]^ Therefore, it is worth paying attentions to whether multiple intravitreal injections of anti-VEGF drugs will affect the cornea. At the same time, some studies have confirmed that bevacizumab can cause appearance of immune markers in the anterior chamber and corneal stroma 1 day after injection,^[6]^ indicating that it can be passively diffused into the corneal stroma and might affect the corneal nerves. So it is speculated that multiple injections of anti-VEGF drugs into the vitreous cavity may affect corneal nerves. Polat et al^[7]^ found that multiple intravitreal injections of anti-VEGF had an effect on the subbasement nerve fiber plexus (SBNP) of the cornea and also reduced the corneal sensitivity. However, some scholars put forward different opinions^[8]^ and found that multiple intravitreal injections of anti-VEGF drugs had no significant effects on the SBNP. Most of the literatures are retrospective studies, and the numbers of intravitreal injections are no more than 3 times. Also, most observations focus on only 1 disease with a single drug, and have no same conclusions. The drugs studied in the literature are mainly Bevacizumab and Ranibizumab, and seldom Aflibercept. So, according to above, we conducted this prospective study to observe the effects of multiple intravitreal injections of Ranibizumab and Aflibercept on the corneal nerves of patients with nAMD, RVO and DME, in order to provide reference data for clinical treatment.

## 2. Materials and methods

Prospective cohort study. A total of 64 patients (64 eyes) who received intravitreal injections of anti-VEGF drugs in the outpatient department of our hospital from September 2021 to June 2022 were selected. There were 33 males and 31 females, with an average age of 57 ± 8.065 years. There were 20 DME cases, 19 nAMD cases and 25 RVO cases. Twenty-nine cases were treated with Aflibercept (2 mg/0.05 mL) and 35 cases with Ranibizumab (0.5 mg/0.05 mL). All enrolled patients were administered monocular injection and followed up with 3 + PRN (pro re nata). Sixty-four patients received 3 consecutive injections; 10 patients received 4 consecutive injections. This study was conducted in accordance with the Declaration of Helsinki and was approved by the hospital’s ethics review committee.

Inclusion criteria: Clinically confirmed cases of nAMD, DME, RVO, and anti-VEGF therapy was required. Receiving intraocular injection for the first time. No history of contact lens. No history of refractive surgery or eye surgery. No history of ocular laser treatment within 3 months before injection. Exclusion criteria: Any corneal/ocular surface disease. Have contact lens history. Previous history of trauma, ocular laser and ocular surgery. Associated with other retinal or optic nerve diseases.

The various clinical examinations included bested corrected vision acuity, intraocular pressure, slit-lamp examination, and fundus ophthalmoscopy. Optical coherence tomography was performed to confirm the diagnosis of macular edema and to evaluate the treatment response. The corneal SBNP was examined by using confocal microscope (Heidelberg) with a red laser wavelength of 670 nm. Different locations of the whole cornea were photographed, and images of SBNP within 6 mm of the central subepithelial area of the cornea were selected for Image J software analysis. The same experienced inspector operated each time, and 3 pictures with the clearest SBNP image were selected for data analysis, and the data were averaged to reduce the error caused by unclear images or slight differences in the selection position. The average amplification was about 800 times. The shooting range was 400 μm × 400 μm (384 pixels × 384 pixels).

Confocal microscopy test was performed by the same experienced inspector. The patient was examined and photographed at the various positions of the whole cornea (central, upper, lower, nasal, and temporal). Finally, the corneal nerve fiber images within 6 mm of the corneal subepithelial central region were selected and processed by Image J software. Image J software analysis included: measurement of cornea nerve fiber length: the total length of nerve fibers per square millimeter; cornea nerve fiber density: the number of nerve fibers per square millimeter.

The eyes were anesthetized with promethacaine (ALCON), 1 drop/5 min, for 3 consecutive times. Povidone iodine was then used to soak the conjunctival sac for 1 minute, and the drug was injected into the vitreous with a 30-gauge needle from 3.5 to 4 mm behind the corneal limbal. Levofloxacin (Santen) eye drops were used 4 times a day for 1 week after operation.

The data related to baseline and 1 month after each injection were collected, including bested corrected vision acuity, intraocular pressure, slit lamp, fundus microscope and optical coherence tomography to evaluate the therapeutic effect and design the next treatment plan. In addition, corneal confocal microscopy was performed to evaluate the influence of intravitral injection of anti-VEGF drugs on the corneal nerve. All cases has been follow up at least 6 months.

### 2.1. Statistical analysis

Statistical analysis was performed by using SPSS 22.0. Shapiro–Wilk conducts the normality test. Mean ± standard deviation was used for the normal distribution data and independent t test was employed for comparison between the 2 groups. The paired t test was used for the comparison between 2 groups, ANOVA was used for comparison between the multiple groups, repeated measurement data was used for repeated measurement ANOVA, and LSD method was employed for the multiple comparison. The non-normal distribution data was represented by median (quartile). Mann–Whitney nonparametric test was used for comparison between the 2 groups, Wilcoxon signed rank sum test was used for comparison between the 2 groups in paired design, and Kruskal–Wallis nonparametric test was used for comparison between the multiple groups.

## 3. Results

**Figure 1.**
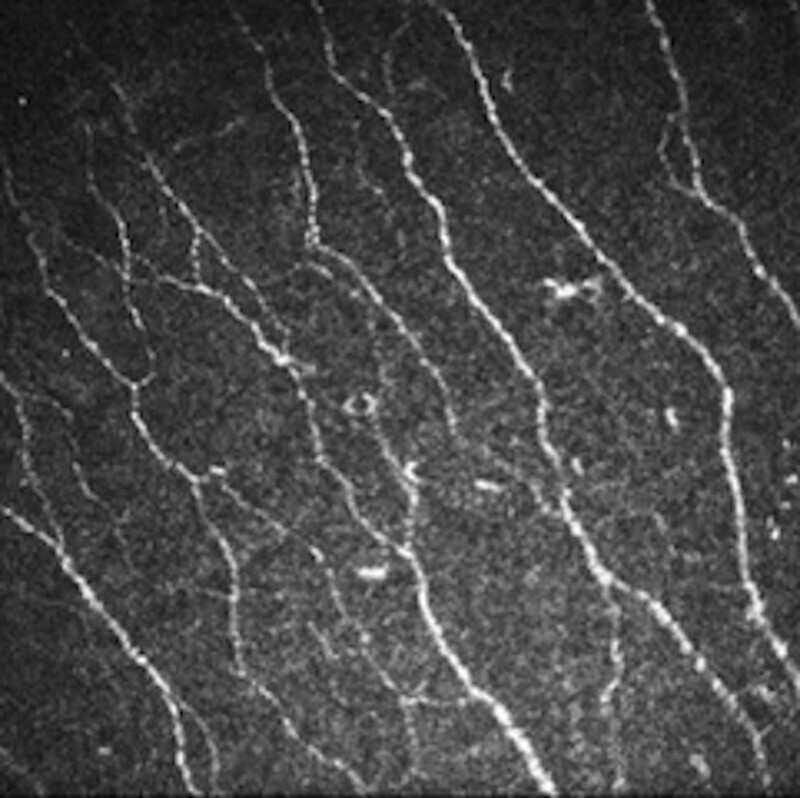
The SBNP image of baseline (contralateral eye). SBNP = subbasal nerve plexus.

**Figure 2.**
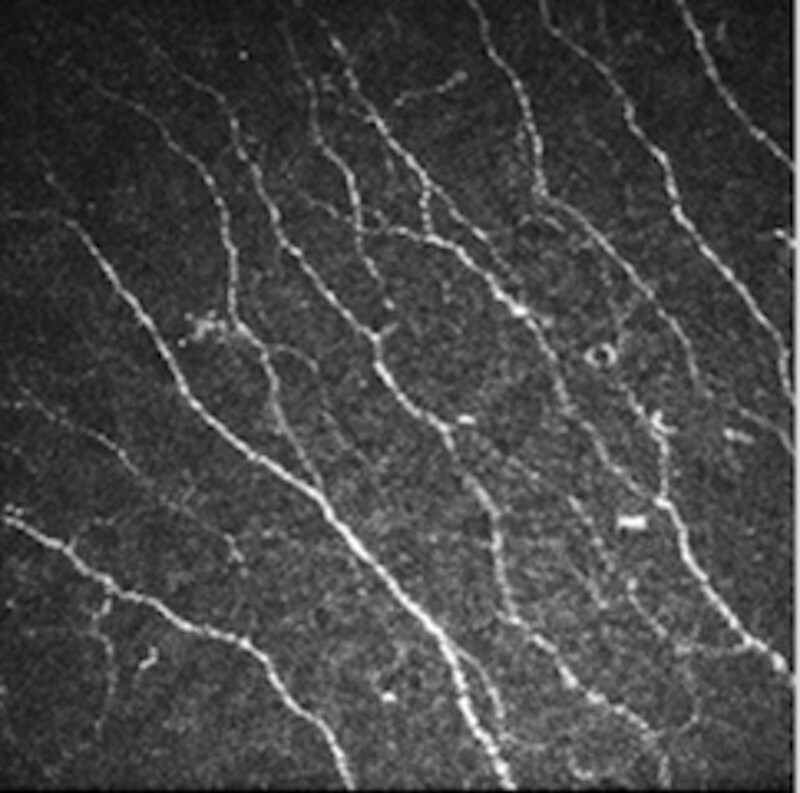
The SBNP image after 3rd injection (contralateral eye). SBNP = subbasal nerve plexus.

Changes of density and length of the corneal nerve fibers after multiple injections in the operative eye and contralateral eye compared with the baseline. It was found that in patients who were administered 3 times of intravitreal injections, the density and length of corneal nerve fibers showed no significant difference at each time after the surgery compared with baseline both in operative eye and contralateral eye (Table 1; Figs. 1 and 2).

**Table 1:** Changes of indexes after 3 injections compared with baseline (operative eye and contralateral eye).

Group	Index	Baseline (n = 64)	After 1st (n = 64)	After 2nd (n = 64)	After 3rd (n = 64)	*F*	*P*
Operative (n = 64)	Nerve density (no./mm^2^)	47.17 ± 16.14	47.69 ± 13.71	46.58 ± 10.84	45.25 ± 9.26	0.944	.379
	Nerve length (mm/mm^2^)	126.51 ± 43.47	126.08 ± 44.61	121.57 ± 36.83	118.08 ± 34.28	2.612	.076
Contralateral (n = 64)	Nerve density (no./mm^2^)	47.17 ± 14.85	47.45 ± 12.49	47.13 ± 9.66	48.59 ± 7.56	0.342	.694
	Nerve length (mm/mm^2^)	117.58 ± 45.43	118.55 ± 41.24	120.64 ± 40.02	119.92 ± 36.65	0.244	.714

Changes of corneal indexes after different numbers of injections. After administration of different numbers of injections, there was no significant differences observed in corneal nerve indexes of the operative eye, and the injection number was irrelevant to the changes in corneal nerve (Table 2).

**Table 2:** Comparison of various indexes of eyes with different injection numbers.

Index	Mean ± SD		*t*/*z*	*P*
	After 3 injections (n = 54)	After 4 injections (n = 10)		
Nerve density (number/mm^2^)	43.750 (42.25, 50.00)	43.750 (35.94, 45.31)	−1.318	.187
Nerve length (mm/mm^2^)	125.215 (102.01, 148.63)	99.690 (75.28, 127.66)	−1.729	.084

Fifty-four patients received 3 consecutive injections, 10 patients received 4 consecutive injections.

Changes in corneal nerve indexes after multiple injections in the operative eye compared to baseline between different diseases.

The corneal nerve indexes at the baseline of operative eyes with different diseases was compared. There were no significant differences observed in the corneal nerve density and nerve length of patients with 3 different diseases at the baseline (Table 3).

**Table 3:** Changes of corneal nerve before and after injection in patients with different diseases.

Group	Number	Nerve density (number/mm^2^)		Nerve length (mm/mm^2^)	
		Baseline [M (P25, P75)]	After 3rd [M (P25, P75)]	Baseline (mean ± SD)	After 3rd [M (P25, P75)]
DME	20	40.625 (37.50, 48.44)	43.750 (37.56, 43.75)	116.26 ± 39.15	104.120 (77.23, 120.70)‡
nAMD	19	50.000 (43.75, 62.50)	43.750 (43.75, 50.00)	133.86 ± 38.07	135.120 (109.89, 144.66)†
RVO	25	43.750 (31.25, 62.50)	43.750 (37.50, 53.13)	129.11 ± 50.23	137.690 (98.69, 158.89)†
*F*/χ^2^		3.517	3.066	0.868	8.200
*P*		.172	.216	.425	.017*

DME = diabetic macular edema, nAMD = age-related macular degeneration, RVO = retinal vein occlusion.

* *P* ≤ .05.

† *P* < .05 vs DME.

‡ *P* < .05 vs nAMD.

The changes of corneal nerve indexes with different diseases after multiple injections were then compared. After administration of injection for 3 contrasting times, the nerve length in DME group was markedly shorter than that in AMD group as well as RVO group, and the difference was statistically significant (*P* < .05). There were no significant differences noted in the nerve density in DME group compared with AMD group and RVO group. There were no significant differences in nerve length and nerve density between the AMD group and the RVO group (Table 3).

Changes in corneal nerve indexes after multiple injections in the operative eye compared to baseline between different drugs.

The changes of corneal nerve indexes after multiple intravitreal injections of Aflibercept were compared. The corneal nerve length after the 2nd and 3rd injections were shorter than that before surgery, the difference was statistically significant (*P* < .01), and the difference of nerve density at each time point was not statistically significant (Table 4).

**Table 4:** Comparisons in patients with aflibercept and ranibizumab after 3 injections (operative eye).

Group	Index	Baseline	After 1st	After 2nd	After 3rd	*F*	*P*
Aflibercept (n = 29)	Nerve density (no./mm^2^)	49.35 ± 15.07	47.62 ± 13.40	45.90 ± 10.72	45.26 ± 8.94	1.964	.150
	Nerve length (mm/mm^2^)	133.77 ± 39.24	122.18 ± 48.68	118.32 ± 39.39†	114.64 ± 36.13†§	5.687	.006*
Ranibizumab (n = 35)	Nerve density (no./mm^2^)	45.36 ± 16.97	47.74 ± 14.16	47.14 ± 11.07	45.24 ± 9.64	0.587	.517
	Nerve length (mm/mm^2^)	120.49 ± 46.38	129.31 ± 41.38	124.26 ± 34.91	120.93 ± 32.93	1.481	.235

* *P* ≤ .01.

† *P* < .05 vs baseline.

‡ *P* < .05 vs “After 1st”.

§ *P* < .05 vs “After 2nd”.

**Figure 3.**
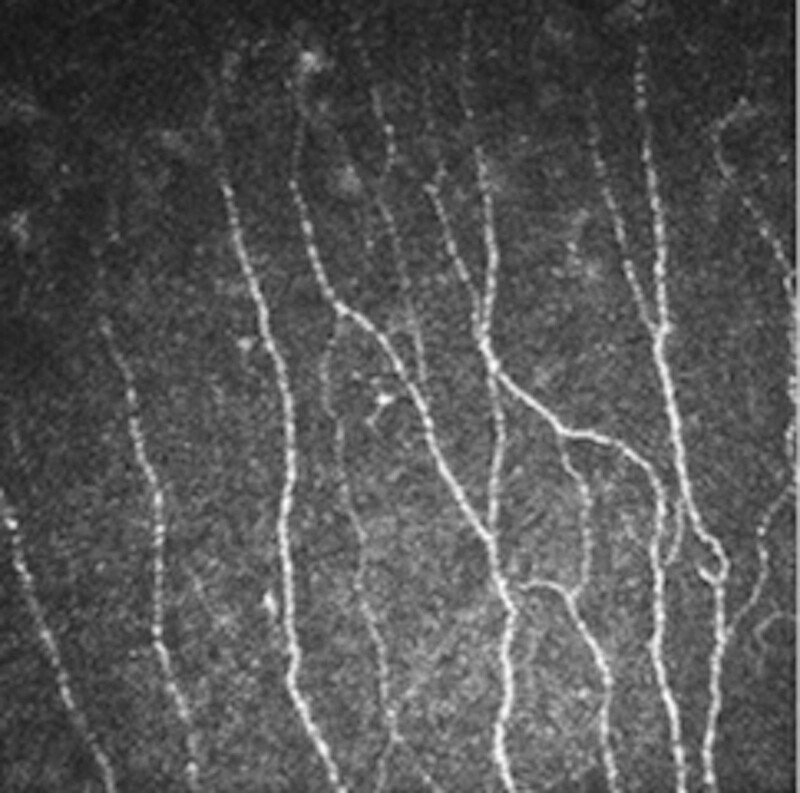
The SBNP image of baseline (operative eye). SBNP = subbasal nerve plexus.

**Figure 4.**
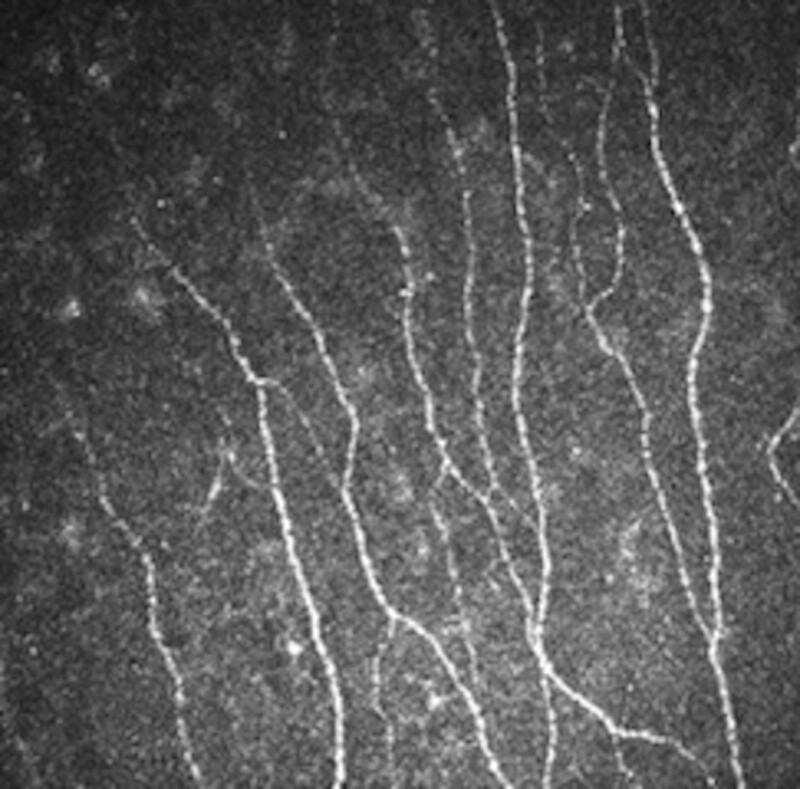
The SBNP image after 3rd injection (operative eye). SBNP = subbasal nerve plexus.

The changes of corneal nerve indexes after multiple intravitreal injections of Ranibizumab were compared. By comparing the changes of corneal nerves at different time points of the eyes after Ranibizumab injection, there were no significant differences in nerve density and nerve length at each time point (Table 4; Figs. 3 and 4).

Compared the level of corneal nerve before injection of the 2 drugs, the difference of nerve density and nerve length at baseline was not statistically significant, so it was comparable (Table 5).

**Table 5:** Comparison of various indexes in patients with different drugs (operative eye).

	Index	Mean ± SD		*t*/*z*	*P*
		Aflibercept (n = 29)	Ranibizumab (n = 35)		
Baseline	Nerve density (number/mm^2^)	43.750 (40.63, 53.13)	37.500 (31.25, 56.25)	−1.52	.129
	Nerve length (mm/mm^2^)	133.77 ± 39.24	120.49 ± 46.38	1.222	.226
After 3rd	Nerve density (number/mm^2^)	43.75 (43.75, 46.88)	43.75 (37.50, 50.00)	−0.275	.078
	Nerve length (mm/mm^2^)	118.450 (79.74, 145.39)	118.450 (103.12, 147.87)	−0.62	.535

Compared the level of corneal nerve after injections of the 2 drugs, the difference of nerve density and nerve length at each point after injections was not statistically significant (Table 5).

At the baseline, there was no statistically significant difference found in cornea indexes between the contralateral eye and operative eye (Table 6).

**Table 6:** Comparison of baseline indexes between operative eye and contralateral eye.

Index	Mean ± SD		Difference (operative-contralateral)	*t*/*z*	*P*
	Operative eye (n = 64)	Contralateral eye (n = 64)			
Nerve density (number/mm^2^)	47.17 ± 16.14	47.17 ± 14.85	−0.00	−0.105	.916
Nerve length (mm/mm^2^)	126.51 ± 43.47	117.58 ± 45.43	8.93	1.704	.093

Complications: All patients were followed up 3 to 6 months after surgery. There were no systemic or ocular adverse events reported during this period.

## 4. Discussion

In recent years, anti-VEGF drugs have been widely employed to treat fundus diseases such as nAMD, DME, and RVO, and have exhibited promising results.^[9–11]^ Although it has been reported previously that intravitreal injection of anti-VEGF drugs might lead to complications such as vitreous hemorrhage, endophthalmitis and intraocular pressure increase,^[12]^ there are few reports describing the possible influence of corneal nerve.

The cornea is the most densely distributed nerve tissue in the human body, mainly innervated by the eye branch of the trigeminal nerve, which then forms the subepithelial nerve plexus in the corneal stroma, whereas its ascending branch passes through the front elastic layer and terminates extensively in the surface cortex.^[13,14]^ The complete distribution of the various corneal nerves plays a key role in the regulation of the normal blink reflex, the stability of epithelial microenvironment, and the distribution and secretion of tears.^[15]^ Corneal confocal microscopy is a method used for the quantitative assessment of corneal nerve morphology. It provides real-time visualization of the corneal nerve fibers in a noninvasive manner and obtained corneal images can be analyzed with appropriate software to identify the quantitative indexes. In our prospective study, we obtained SBNP images using corneal confocal microscopy and analyzed the data using Image J software.

VEGF-A, VEGF-B, VEGF-C, VEGF-D, VEGF-E and placental growth factor, belong to the VEGF family, and act as indispensable key factors in regulating the process of angiogenesis. They can function to induce angiogenesis by stimulating mitosis and migration of the vascular endothelial cells. VEGF family has been found to play a vital role in the development and maintenance of the blood vessels and nerves, as well as in modulating the processes of neuro-nutrition and neuroprotection.^[16]^ Marfurt et al^[17]^ reported that VEGF-A165B, subtype of VEGF-A, can exhibit neuro-protective and anti-damage effects on epidermal sensory neurons in the diabetic rats, and experimental data also indicated the neuro-protective effects of VEGF on the retinal nerve cells.^[18]^ Therefore, it can be speculated that anti-VEGF drugs might be unfavorable for nerve protection and nerve nutrition. Muchen et al^[19]^ examined the subconjunctival injection of bevacizumab in the normal mice and found that bevacizumab can significantly cause corneal nerve decline, sensory dullness, delayed epithelial repair, and affect corneal nerve regeneration, and the greater the concentration of bevacizumab used and the number of injections, the more obvious the impact. Polat et al^[7]^ employed confocal microscopy to observe the effects of multiple intravitreal injections of anti-VEGF on the corneal nerve fibers and corneal sensitivity and observed that multiple intravitreal injections of anti-VEGF had marked effect on SBNP, and could also significantly reduce the corneal sensitivity.

Our study found that after multiple intravitreal injections of anti-VEGF drugs, there were no statistically significant differences in both the density and length of corneal nerve fibers at each postoperative time compared with baseline, which was similar to the findings of Gulfidan et al.^[17]^ In his study, 33 patients with nAMD (66 eyes) were injected once a month into the vitreous for at least 3 months. The control group was selected from the non-injected eyes and few healthy subjects and there was no statistical difference found in corneal perception and SBNP between the control group and the experimental group. In another study, Bock F reported based on the findings of the preclinical experiments^[8]^ that bevacizumab did not affect the corneal structure, nerve repair or nerve fiber density in the normal mice.

We also compared the corneal nerve conditions in patients with 3 different diseases. At baseline, the corneal nerve density and nerve length in DME group were observed to be shorter than those in AMD group and RVO group, but the difference was not statistically significant. After multiple injections, the nerve length of DME group was also shorter than that of AMD group as well as RVO group, and the difference was statistically significant, but the nerve length and nerve density of AMD group and RVO group were not statistically significant after multiple injections. It has been reported that diabetic patients are more likely to show progressive loss of corneal nerve fibers and decreased corneal sensitivity in comparison to those affected with other diseases.^[21]^ Therefore, multiple injections of anti-VEGF drugs are more likely to cause corneal nerve changes in the diabetic patients. A study^[22]^ reported that corneal nerve fiber length was more sensitive relative to the nerve density in diabetic patients with/without the development of diabetic retinopathyetinopathy for 4 years and was one of the main predictors of diabetic retinopathyetinopathy deterioration. Therefore, in this study, after multiple injections of anti-VEGF drugs, the length of corneal nerve in diabetic patients was affected only to a certain extent, but the corneal nerve density was not significantly affected. Although, the corneal nerve fiber density and length in DME patients were shorter than those in nAMD and RVO patients at baseline, the differences were not statistically significant. But after the third intravitreal injection, the nerve length of DME patients was lower than that of nAMD and RVO patients. Diabetes mellitus is a risk factor affecting the corneal nerve. We also compared the corneal nerve length after the 3rd injection of contralateral eye (non-injected eye) (112.09 ± 44.54) in DME group with baseline (113.28 ± 30.39), and there is no significant difference (t = -0.118 *P* = .907), indicating that the reduction of corneal nerve length in operative eye was not caused by the natural course of diabetes mellitus, but mainly by drugs.

At the same time, we also compared the effects of 2 different drugs (Aflibercept and Ranibizumab) on the corneal nerve. We found that corneal nerve length after the 2nd and 3rd injections of Aflibercept was shorter than that before surgery, and the difference was statistically significant. There were no significant differences in nerve density and nerve length at different time points after multiple injections of Ranibizumab. Therefore, compared with Ranibizumab, the injection of Aflibercept may have a greater effect on corneal nerve length. We know that intravitreal injection of 2 mg Aflibercept had a molar concentration of 348 nmol/mL and 0.5 mg Ranibizumab had a molar concentration of 208 nmol/mL, and Aflibercept also had a longer vitreous activity than Ranibizumab, so we speculated that Aflibercept had a higher molar concentration and longer activity which making its potential beneficial on macular edema and adverse effects on corneal nerve more stronger than Ranibizumab.

Interestingly, studies have shown that after monocular injection of anti-VEGF drugs, they can effectively enter the contralateral eye through the systemic circulation and play a certain role in reducing the thickness of macula and improving vision to a certain extent.^[23,24]^ We considered that anti-VEGF drugs which have a positive effect on the contralateral eye, might also cause significant side effects on contralateral eye. Therefore, in this study, we also observed the changes of the various indexes of corneal nerve fibers in contralateral eyes after the multiple injections. We found that the corneal nerve density and nerve length in the contralateral eye showed no major statistical significance at each time after the multiple intravitreal injections compared with baseline.

It has been reported that delayed wound healing, dry eye, persistent epithelial defect, and neurotrophic corneal ulcer can occur after corneal nerve injury, which might lead to the severe corneal infection, vision loss or even blindness.^[25][Bibr R20]^ Hence, the corneal nerve deserves proper attention, especially for diabetic patients. It is also needed to strengthen the local anti-inflammatory therapy to avoid infection and to use artificial tears to protect the microenvironment of the ocular surface after the surgery.

To the best of our knowledge, the clinical literatures describing the potential effects of multiple injections of anti-VEGF drugs on the cornea nerve are relatively limited, and most of the reported studies have used intravitreal injection for 3 times. In our study, we have also initially explored the influence of intravitreal injected for 4 times, and also compared the corneal nerve indexes in patients with 3 different diseases and different drugs, which is the major innovative aspect. Of course, our study still has few limitations. The follow-up time of patients in this study is short, especially the sample size of patients who were administered 4 injections is relatively small. It is still necessary to enlarge the sample size for further observations. Animal experiments are lacking in this subject. To explore the mechanism of its influence on neural state at the cellular or animal level. We also studied the influence of anti-VEGF on corneal endothelium and corneal biomechanics, and the paper is being written. We also look forward to the clinical application of the drugs in the intraocular drug sustained-release delivery system for the management of retinal vascular diseases.

## Author contributions

**Conceptualization:** Qi Yuanyuan.

**Data curation:** Lin Cui, Li Zhang, Shuang Ye.

**Formal analysis:** Yao Jiang, Shuang Ye.

**Investigation:** Yao Jiang, Lili Ji, Yuanyuan Qiu.

**Methodology:** Chunxiao Yan, Lili Ji, Yuanyuan Qiu.

**Software:** Chunxiao Yan.

**Supervision:** Lijun Zhang.

**Writing – original draft:** Qi Yuanyuan.

**Writing – review & editing:** Lijun Zhang.
